# Uncovering Key Characteristics of Antibacterial Peptides through Machine Learning

**DOI:** 10.1002/marc.202500583

**Published:** 2025-09-28

**Authors:** Jooyoung Roh, Cyrille Boyer, Priyank V. Kumar

**Affiliations:** ^1^ School of Chemical Engineering University of New South Wales (UNSW) Sydney New South Wales Australia; ^2^ Australian Centre for NanoMedicine University of New South Wales (UNSW) Sydney New South Wales Australia

**Keywords:** antibacterial polymers, artificial intelligence, cell structure, classification, gram‐negative, gram‐positive, machine learning, mycobacteria, random forest

## Abstract

Antimicrobial peptides (AMPs) have emerged as promising alternatives to traditional antibiotics in addressing the growing threat of multi‐drug‐resistant (MDR) bacteria—a crisis that could lead to millions of deaths over the next three decades if left unaddressed. While the general role of cationic and hydrophobic interactions in AMP‐mediated bacterial killing is well established, the distinctions between structural characteristics of AMPs targeting different types of bacteria remain underexplored. To address this issue and streamline the design of potent AMPs depending on the bacterial structure, machine learning (ML) models were employed on AMPs targeting Gram‐negative bacteria (*Pseudomonas aeruginosa* PAO1), Gram‐positive bacteria (*Staphylococcus aureus* ATCC 29213), and mycobacteria (*Mycobacterium tuberculosis* H37Rv and *Mycobacterium smegmatis* mc^2^ 155) to derive important features that determine antimicrobial efficacy. The Random Forest models mainly reveal that AMPs with a cLogP of less than ‐6 and a net‐charge limited to +4 with variations of the hydrophobic composition in between 20%–50% (20%–40% against *P. aeruginosa* PAO1, 30%–50% against *S. aureus* ATCC 29213, 35%–45% against *M. tuberculosis* H37Rv and *M. smegmatis* mc^2^ 155) and variations of the cationic composition in between 10%–40% (10%–20% against *P. aeruginosa* PAO1, 30%–40% against *S. aureus* ATCC 29213, 10%–30% against *M. tuberculosis* H37Rv and *M. smegmatis* mc^2^ 155) predicts significant antibacterial activity. The feature characteristics of the three bacterial types may directly relate to their distinct cell envelope structures and aid in mode of action postulation. This work demonstrates how ML can effectively inform AMP design by accounting for microbial structural differences and underscores its broader potential in customizing peptides for specific bacterial strains.

## Graphical abstract

Machine‐learning (ML) techniques using random forest classification models revealed key characteristics that predict effective antimicrobial peptides (AMPs) targeting Gram‐negative bacteria, Gram‐positive bacteria, and mycobacteria. The ideal cLogP (<−6) and net‐charge (≤+4) threshold was the same for all three targets with variations in the hydrophobic (20%–50%) and cationic (10%–40%) compositions in relation to their unique cell‐envelope.

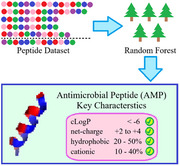

## 1 Introduction

The usage of traditional antibiotics has successfully saved millions of lives from bacterial infections since the discovery of the first antibiotic, penicillin, in the early 20th century; however, its excessive usage has led to antibiotic resistance [1, 2, 3]. Some microbial strains present multidrug resistance (MDR), known as “superbugs,” and are extremely hard to treat with conventional antibiotics and may be responsible for up to 10 million deaths worldwide by 2050 [4, 5, 6, 7]. Alarmingly, some bacteria can evolve at such a pace that resistance can be achieved within one week, but the development of new antibiotics typically takes at least 10 years [8]. Therefore, it is essential to rely on alternatives with bactericidal effects that can specifically target different bacterial types and species without antibiotic resistance concerns. One promising alternative is antimicrobial peptides (AMPs) consisting of cationic and hydrophobic residues, which are short peptides consisting of 10–50 amino acids [9, 10, 11]. AMPs fold into secondary structures such as α‐helix, β‐sheet, β‐hairpin, etc.) via intermolecular forces that adopt an amphiphilic conformation with positively charged and hydrophobic ends [12, 13, 14], which have been recognized for their ability to actively fight against multidrug‐resistant bacteria due to their non‐selective mode of action [10]. Currently, there are more than 3000 AMPs identified by the scientific community, where each has its distinct level of antimicrobial efficacy for different types of microbial species and strains based on their unique peptide sequence [15, 16].

Although the specific mechanism of killing is still under dispute, biocidal activity via membrane perturbation with electrostatic and hydrophobic interactions – inducing cytoplasmic fluid leakage is the broadly accepted theory [17]. Membrane destructive mechanisms include the barrel‐stave model first reported in the 1970s, where AMPs are inserted vertically into the membrane bilayer along the direction parallel to the phospholipid chains, where the cationic and hydrophobic groups align with the head and tail of the lipids, respectively to form pores [18]. The carpet model was introduced in 1992, where after a critical concentration was achieved, the adsorbed AMPs produced a detergent‐like effect to decompose the membrane and form phospholipid micelles; thus, it was named a detergent‐like model [19]. Soon after, the toroidal pore model was proposed, where AMPs induce local membrane bending, resulting in the formation of pore‐lining arranged by peptides and phospholipid head groups [20]. Researchers have more recently proposed nonmembrane destruction mechanisms where the formation of pores or micelles is not necessary to kill bacteria, and disrupting the integrity and function is sufficient. A membrane thinning model has been proposed where cationic peptides are adsorbed on the microbial membrane by electrostatic interaction with anionic lipid head groups, a gap forms in the hydrophobic region, and neighboring peptides tilt to fill the gap since the total volume of the lipid bilayer cannot be changed – resulting in membrane thinning [21, 22]. Contrastingly, a membrane thickening model was more recently proposed, where the authors discovered that if an AMP failed to insert into the bilayer in full length, the phospholipids stretched out to the hydrophobic tails to effectively bind with it, resulting in membrane thickening [23]. More recently, several complex intracellular antibacterial mechanisms have also been proposed, but the process typically also involves membrane perturbation [24, 25, 26, 27].

**FIGURE 1:**
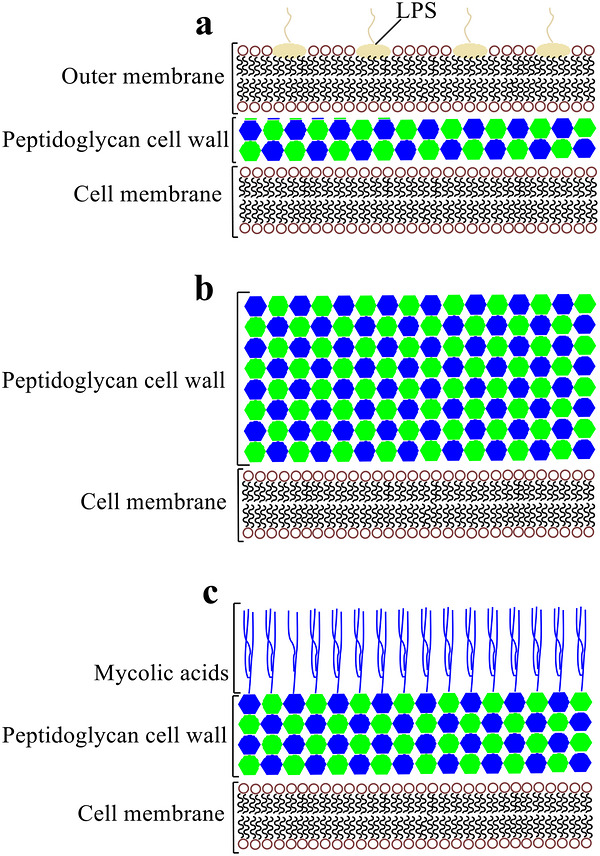
Simplified cell envelope illustrations of (a) Gram‐negative bacteria: consisting of a plasma inner membrane IN, thin peptidoglycan cell wall (>10 nm) and a lipopolysaccharide (LPS) – rich outer membrane (OM). (b) Gram‐positive bacteria: consisting of a plasma IN and thick peptidoglycan cell wall (20–80 nm). (c) Mycobacteria: consisting of a plasma IN, moderately a thick peptidoglycan cell wall (15–20 nm), and mycolic acids.

While the general theory of AMPs killing bacteria relying on their cationic and hydrophobic residues has been substantially established and widely accepted [28], the distinctions between AMPs that effectively target the three different types of bacteria: Gram‐negative bacteria, Gram‐positive bacteria, and mycobacteria, have not yet been deeply investigated. We anticipate differing AMP characteristics between the three types due to their distinct structural features (See Figure 1).

For instance, although all three bacterial microbes share similar cytoplasmic membranes (inner membranes)—composed of zwitterionic and anionic phospholipids that confer a net‐negative surface charge [29]—their structural differences in the other cell envelope components outside the inner membranes likely drive differences in AMP characteristics [30]. The cell wall for Gram‐positive bacteria is composed of thick peptidoglycan layers (20–80 nm), where its stability is further strengthened by wall teichoic acids (WTA) and lipoteichoic acids (LTA), which are electronegative [31]. On the contrary, the cell‐wall thickness for Gram‐negative bacteria is much thinner (∼10 nm) for Gram‐negative bacteria but it gains extra protection from an additional outer membrane (OM), which has highly negatively charged lipopolysaccharides (LPS) [31, 32]. Mycobacteria are protected by a moderately thick cell wall [33] (∼15 nm) with an additional protective outer layer consisting of highly hydrophobic mycolic acids [34, 35, 36]. Due to such structural differences, it is expected that the optimal AMP composition, charge, and hydrophobicity (cLogP) and other qualitative peptide features required for significant antimicrobial activity may align or vary among the four microbes, depending on the target microbe and its respective envelope, as the microbial structure is expected to dictate the main mode of action.

Machine learning (ML) could help unravel these feature similarities and differences given its past success for a variety of applications [37, 38, 39, 40, 41], including polymers [42, 43]. Examples include linear regression models to predict the lower critical solubility temperature of polymers [44], decision trees that makes decisions based on specific nodes [45] for refractive index predictions of polymers [46], and random forests, which involve multiple decision trees to improve their accuracy [47] to predict glass transition temperatures [48]. For biochemical applications specifically, Tiihonen et al. predicted the antimicrobial activity of conjugated oligoelectrolyte molecules (COEs) using ML techniques by using a recursive elimination process to select 21 molecular features originally from 5305 descriptors. The predictive model presented a moderate accuracy with an R^2^ value of 0.65 [49].

More recently, Kundi et al. used a decision tree classification model for antibacterial synthetic polymers to identify that the most important feature in predicting antibacterial efficacy for Gram‐negative bacteria was the cLogP in a range between −2 and +0.5 [50] partially agreeing with previous experimental findings from Boyer and colleagues, that the cLogP was a very strong predictor in antibacterial efficacy (although the ideal values had to be between 0 to 2 as the cLogP calculation methods differed) [51], followed by the cationic composition and hydrophobic composition [52, 53, 54]. However, using ML to identify the important features that predict and determine the antimicrobial efficacy of AMPs has been underexplored. Even though the unique peptide sequence (of a particular amino acid order) ultimately determines the exact antibacterial efficacy of the peptide, ML models that effectively identify the broader key features could significantly streamline the selection of effective AMPs tailored to specific bacterial strains.

In this work, we develop random forest‐based classification models for AMP datasets generated from the DBAASP database [55] by categorizing amino acids into five categories based on their charge and hydropathy based on the Kyte‐Doolittle scale [56] and identify relevant AMP criteria for Gram‐negative (*Pseudomonas aeruginosa* PAO1), Gram‐positive (*Staphylococcus aureus* ATCC 29213), and mycobacterial (*Mycobacterium tuberculosis* H37Rv and *Mycobacterium smegmatis* mc^2^ 155) bacterial strains. To effectively target Gram‐negative *P. aeruginosa* PAO1, the model predicts that AMPs should have cLogP values below ‐6, a hydrophobic composition of 20%–40%, a cationic composition of 10%–20%, and a net charge between 0 and +4. On the other hand, to effectively target Gram‐positive *S. aureus* ATCC 29213, our model predicts that AMPs should exhibit cLogP values below ‐6, hydrophobic composition of 30%–50%, cationic composition of 30%–40%, a net charge of 0 to +4, and include neutral (hydropathy‐wise) amino acids. Notably, to effectively target mycobacteria *M. tuberculosis* H37Rv and *M. smegmatis* mc^2^ 155, the model predicts that AMPs should have cLogP values below −6, a hydrophobic composition of 35%–45%, cationic composition of 10%–30%, net‐charge of +2 to +4, and a peptide length of at least 20. All three bacterial types remarkably have a shared cLogP threshold of less than −6 for predicting significant antimicrobial activity. Our findings also reveal different compositional requirements for AMPs targeting the three different types of bacteria, reflecting their structural differences. This study highlights the utility of ML in guiding AMP design based on bacterial architecture, with potential for broader application in tailoring peptides to specific microbial species and strains.

## 2 Methods

### 2.1 Computational Details

All ML studies were carried out using Python programming in the Jupyter notebook environment, and the packages used in this work were Pandas [57, 58], Numpy [59], Scikit‐Learn [60], RDKit [61], and Shapley Additive exPlanations (SHAP) [62]. The codes used in the study are available in the Data Availability section in the Supporting Information.

### 2.2 Data Acquisition and Feature Engineering

Datasets containing more than 300 datapoints were generated based on the DBASSP database [51]. The target species was set to “Pseudomonas aeruginosa PAO1” (325 datapoints of AMPs targeting *P. aeruginosa* PAO1), “*Staphylococcus aureus* ATCC 29213” (395 datapoints of AMPs targeting *S. aureus* ATCC 29213), and “Mycobacterium tuberculosis H37Rv” and “Mycobacterium smegmatis mc^2^ 155” (80 datapoints of AMPs targeting *M. tuberculosis* H37Rv and *M*. *smegmatis* mc^2^ 155) to obtain peptides and their respective minimum inhibitory concentrations to inhibit 90% of bacterial growth (MIC_90_). Only peptides with an MIC_90_ calculated in µg/mL units were included in the dataset to ensure an accurate reflection of the models and eliminate calculation error concerns from unit conversions. Only peptides provided with a Simplified Molecular Input Line Entry System (SMILES) were considered. This was deemed essential as the cLogP was calculated using Python RDKit from the SMILES string. For accurate analysis, all datasets were restricted to peptides with a net charge between 0 and +15. The datasets were also restricted to peptides with a cLogP between −15 and +5 for AMPs targeting *P. aeruginosa* PAO1 and AMPs targeting *S. aureus* ATCC29213, whereas it was restricted to peptides with a cLogP of −15 to 0 for AMPs targeting *M. tuberculosis* H37Rv and *M. smegmatis* mc^2^ 155 for accurate and elaborate analysis.

### 2.3 Amino Acid Type

**FIGURE 2:**
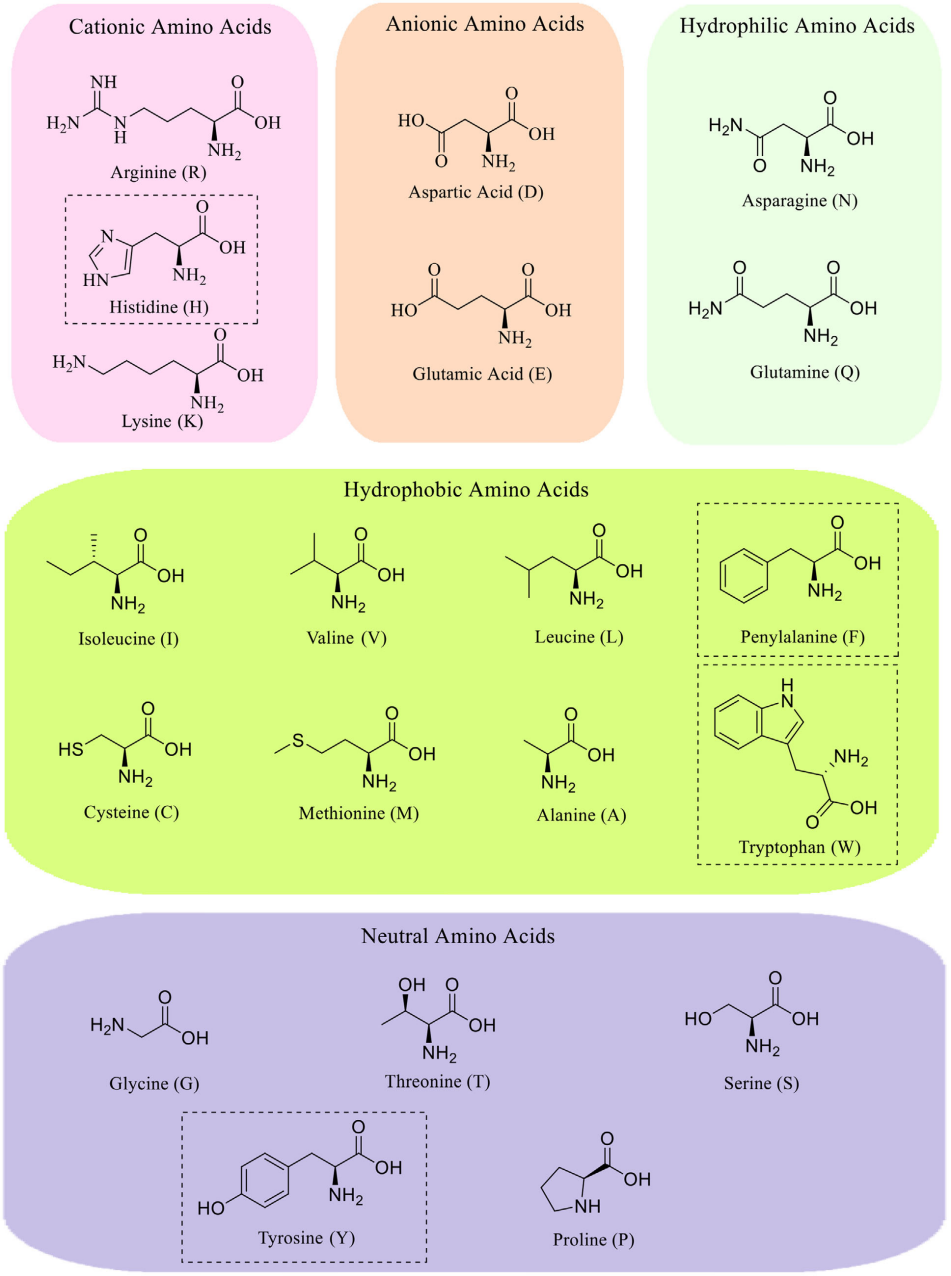
Amino acids are categorized based on their charge and hydropathy. Hydrophilic and neutral amino acids in their hydropathy that were charged (arginine, histidine, lysine, aspartic acid, and glutamic acid) were grouped into the cationic or anionic categories depending on their charge, and not grouped into their respective hydropathy category. Histidine (H), Phenylalanine (F), Tryptophan (W), and Tyrosine (Y) were also assigned to the aromatic category (dotted lines) for additional qualitative analysis.

The amino acids were categorized into five categories based on their charge and hydropathy on the Kyte‐Doolittle scale (See Figure 2) [56]. In terms of hydropathy, eight amino acids with a hydropathy of −0.9 (the hydropathy of tryptophan) or higher were considered hydrophobic, and six amino acids were considered hydrophilic if the hydropathy was −3.5 (the hydropathy of asparagine) or lower. The other six amino acids with moderate hydropathies were considered neutral. The charge took precedence for categorization to effectively understand the effects of cationic composition and inclusion of anionic amino acids and their relation to the net charge of the peptide. This was deemed appropriate as all five of the charged amino acids were one of the seven most hydrophilic amino acids, hence similar in their hydropathy, and there was no hydrophilic feature used for machine‐learning analysis.

For example, Arginine (R) is cationic and hydrophilic, but categorized as cationic but not hydrophilic category. Aspartic Acid (D) is anionic and hydrophilic but categorized as anionic, but not hydrophilic. This was done to clearly understand the influence of the cationic composition, the inclusion of anionic amino acids, and net‐charge on antibacterial efficacy. Based on this rule, three amino acids are categorized as cationic: Arginine (R), Histidine (H), and Lysine (K). Two amino acids were classified as anionic: Aspartic Acid (D) and Glutamic Acid (E): If the hydropathy was at least −0.9 (the hydropathy of tryptophan), it was categorized as hydrophobic. If the hydropathy was −3.5 or lower and had no charge, it was classified as hydrophilic. Based on this rule, two amino acids were categorized as hydrophilic: Asparagine (N) and Glutamine (Q). Five amino acids with a hydropathy greater than −3.5 and less than −0.9 with no charge were categorized as neutral: Glycine (G), Threonine (T), Serine (S), Tyrosine (Y), and Proline (P). Eight amino acids with a hydropathy of at least ‐0.9 were categorized as hydrophobic: Isoleucine (I), Valine (V), Leucine (L), Phenylalanine (F), Cysteine (C), Methionine (M), Alanine (A), and Tryptophan (W). Histidine (H), Phenylalanine (F), Tryptophan (W), and Tyrosine (Y) were also categorized as aromatic amino acids for additional qualitative analysis.

### 2.4 Features

Based on the assignments described above, eight chemical descriptors/features were considered: (1) net‐charge: the overall charge of the peptide obtained directly from the DBAASP database, (2) cLogP: the cLogP value calculated from the RDKit from Python, (3) cationic composition: the number of amino acids belonging to the cationic category divided by the peptide length, (4) hydrophobic composition: the number of amino acids belonging to the hydrophobic category divided by the peptide length, (5) peptide length: the number of amino acids in a peptide sequence, (6) has_aromatic: indicating the presence of aromatic rings; a qualitative feature where a “1” is assigned for a peptide with at least one amino acid being Histidine (H), Phenylalanine (F), Tyrosine (Y) or Tryptophan (W). A “0” is assigned for the feature otherwise, 7) has_neutral: indicating the presence of neutral amino acids; a qualitative feature where a “1” is assigned for the feature if a peptide with an amino acid with at least one amino acid belonging to the neutral category in the sequence. A “0” is assigned for the feature otherwise, and 8) has_anionic: indicating the presence of anionic amino acids; a qualitative feature where a “1” is assigned for the feature if a peptide with an amino acid with at least one amino acid belonging to the anionic category in the sequence. A “0” is assigned for the feature otherwise.

### 2.5 Target (Minimum Inhibitory Concentration)

A peptide was considered to be effective against bacterial activity if its MIC_90_ (minimum inhibitory concentration to inhibit 90% of bacterial growth) was less than or equal to 64 µg mL^−1^. If the MIC_90_ value was a range, the higher value was used as the value for analysis, as it represented the worst‐case scenario, ensuring that peptides with an antimicrobial efficacy that was strictly on or under the designated threshold would only be assigned as effective peptides. For example, if a particular peptide sequence presented a MIC_90_ value range of 64–128 µg mL^−1^, it would be assumed to have an MIC_90_ of 128. Based on the MIC_90_ values, AMPs were divided into two classes for the ML classification models. Antibacterial AMPs, which exhibited a MIC_90_ of 64 µg mL^−1^ or less, were classified as Class 1 (good peptides that can effectively kill bacteria), and AMPs, which exhibited an MIC_90_ of 128 or greater, were classified as Class 0 (poor peptides that fail to effectively kill bacteria). Although tighter thresholds could be used (e.g., MIC_90_ of 16 or 32 µg mL^−1^), 64 µg mL^−1^ was selected to have enough datapoints for Class 1, which is essential for ML model accuracy. The successive standardization and splitting of the three data sets were performed under these rules.

### 2.6 Standardization and Splitting the Data Set

Most ML models require that a data set be standard. Model performance is expected to improve if individual features resemble standard normally distributed data. Therefore, the final data set was standardized using the StandardScalar module from the Scikit‐learn library. To take class imbalance in the data set into consideration, where the number of Class 1 AMPs does not match the number of Class 0 AMPs, a stratified train‐test split approach was employed. This method maintains the proportions of the classes in both the test set and training data sets. This was done to eliminate class bias for ML performance assessment. The stratified split was performed using the train_test_split function available in the Scikit‐learn library, with a ratio of 75% for training data and 25% for testing data.

### 2.7 Classification Models

An extensive examination of seven different classification models, including the Logistic Regression Classifier (LR), C‐Support Vector Classifier (SVC), Naive Bayes Classifier (NBC), Multi‐Layer Perception Classifier (MLP), Decision Tree Classifier (DTC), Random Forest Classifier (RFC), and K‐Nearest Neighbor Classifier (KNN). These models were selected with the intention to apply the best performing ML model for SHAP analysis after assessing their performance.

### 2.8 Selection of the Appropriate Metric

Since Class 1 AMPs are defined as the “good” polymers where the MIC_90_ is 64 µg mL^−1^ or below, it is important for the Class 1 AMPs not to be misclassified as Class 0, as they are the AMPs of interest that will fast‐track the selection of appropriate peptides to kill a certain bacterial strain(s) of interest. If misclassified among Class 0 polymers, valuable insights into the features and characteristics that define effective polymers may be lost, hindering proper analysis. Hence, along with model accuracy, which measures the overall correct predictions, the recall and F1‐score for true positives of Class 1 AMPs are paramount for model performance assessment. The recall assesses how well the true positives of the model are identified compared to false negatives. The precision assesses how well the true positives of the model are identified compared to the false positives, and the F1‐score balances recall and precision for a broader view of parameter performance.

### 2.9 Hyperparameter Optimization

The parameters of the seven ML models were obtained from hyperparameter optimization by using the RandomizedSearchCV technique from the “model selection” module of the Scikit‐Learn Python package. Hyperparameter optimization using the “train” set was performed with repeated stratified k‐fold cross‐validation with 9 splits and 10 repeats for the two datasets of AMPs targeting *P. aeruginosa* PAO1 (Gram‐negative) and *S. aureus* ATCC 29213 (Gram‐positive), respectively. For the dataset of AMPs targeting *M. tuberculosis* H37Rv and *M. smegmatis* mc^2^ 155 (mycobacteria), hyperparameter optimization using the “train” set was performed with repeated stratified k‐fold cross‐validation with 4 splits and 5 repeats. The relatively smaller split and repeats for the dataset of AMPs targeting mycobacteria are to account for its smaller data set of only 80 datapoints, as opposed to the other two relatively larger data sets with more than 300 datapoints. A random state of 0 and 100 iterations was used for all three datasets during hyperparameter optimization. Since having a high recall and F1‐score for Class 1 AMPs is a priority over high accuracy and precision, the scoring metric for hyperparameter tuning of the ML models was set to the recall and F1‐score metrics to obtain potentially high performing parameters of interest (See Tables S1–S3). Then the accuracy, F1‐score, recall, and precision of the hyperparameters were assessed on the “test” set to validate the superior ML model and its hyperparameter for SHAP analysis (See Tables 1, 2, 3, 4, 5, 6). The accuracy, F1‐score, recall, and precision of the hyperparameters were also assessed on the “train” set to further verify the reliability of the classification models for each target microbe (See Tables S4–S9).

**TABLE 1:** Performance summary of all classification models when scoring is set to recall for amps targeting *P. aeruginosa* PAO1. The Random Forest Classifier, highlighted in bold, is chosen as the best‐performing model given its well‐balanced performance across different metrics.

Models	Accuracy	F1	Recall	Precision
**Random Forest**	**0.82**	**0.86**	**0.87**	**0.85**
Decision Tree	0.73	0.81	0.85	0.77
Multi‐Layer Perception	0.66	0.79	1.0	0.66
C‐Support Vector	0.72	0.78	0.74	0.82
Logistic Regression	0.73	0.79	0.76	0.82
Naïve Bayes	0.76	0.82	0.83	0.80
K‐Nearest Neighbors	0.76	0.84	1.0	0.73

**TABLE 2:** Performance summary of all classification models when scoring is set to the F1‐score for amps targeting *P. aeruginosa* PAO1. All models were outperformed by the Random Forest Classifier when scoring is set to recall (See Table 1).

Models	Accuracy	F1	Recall	Precision
Random Forest	0.80	0.85	0.87	0.84
Decision Tree	0.74	0.80	0.78	0.82
Multi‐Layer Perception	0.80	0.85	0.85	0.85
C‐Support Vector	0.73	0.80	0.81	0.79
Logistic Regression	0.73	0.79	0.76	0.82
Naïve Bayes	0.74	0.81	0.83	0.79
K‐Nearest Neighbors	0.77	0.83	0.89	0.79

**TABLE 3:** Performance summary of all classification models when scoring is set to recall for AMPs targeting *S. aureus* ATCC 29213. All models were outperformed by the Random Forest Classifier when scoring is set to F1‐score (See Table 4).

Models	Accuracy	F1	Recall	Precision
Random Forest	0.71	0.77	0.80	0.74
Decision Tree	0.69	0.74	0.74	0.75
Multi‐Layer Perception	0.62	0.76	1.0	0.62
C‐Support Vector	0.63	0.69	0.67	0.71
Logistic Regression	0.66	0.71	0.67	0.75
Naïve Bayes	0.65	0.69	0.64	0.75
K‐Nearest Neighbors	0.67	0.74	0.79	0.71

**TABLE 4:** Performance summary of all classification models when scoring is set to F1‐score for AMPs targeting *S. aureus* ATCC 29213. The best performing model across different metrics (Random Forest Classifier) is highlighted in bold.

Models	Accuracy	F1	Recall	Precision
**Random Forest**	**0.72**	**0.78**	**0.82**	**0.75**
Decision Tree	0.69	0.74	0.72	0.76
Multi‐Layer Perception	0.66	0.73	0.77	0.70
C‐Support Vector	0.66	0.72	0.70	0.73
Logistic Regression	0.66	0.71	0.67	0.75
Naïve Bayes	0.65	0.69	0.64	0.75
K‐Nearest Neighbors	0.68	0.75	0.77	0.72

**TABLE 5:** Performance summary of all classification models when scoring is set to recall for AMPs targeting *M. tuberculosis* H37Rv and *M. smegmatis* mc^2^ 155. All models were outperformed by the Random Forest Classifier when scoring is set to F1‐score (See Table 6).

Models	Accuracy	F1	Recall	Precision
Random Forest	0.70	0.75	0.75	0.75
Decision Tree	0.75	0.82	0.75	0.90
Multi‐Layer Perception	0.60	0.75	1.0	0.60
C‐Support Vector	0.65	0.72	0.75	0.69
Logistic Regression	0.55	0.64	0.67	0.62
Naïve Bayes	0.65	0.74	0.83	0.67
K‐Nearest Neighbors	0.75	0.78	0.75	0.82

**TABLE 6:** Performance summary of all classification models when scoring is set to F1‐score for AMPs targeting *M. tuberculosis* H37Rv and *M. smegmatis* mc^2^ 155. The best performing model across different metrics (Random Forest Classifier) is highlighted in bold.

Models	Accuracy	F1	Recall	Precision
**Random Forest**	**0.80**	**0.83**	**0.83**	**0.83**
Decision Tree	0.75	0.78	0.75	0.82
Multi‐Layer Perception	0.65	0.72	0.75	0.69
C‐Support Vector	0.60	0.69	0.75	0.64
Logistic Regression	0.55	0.64	0.67	0.62
Naïve Bayes	0.65	0.74	0.83	0.67
K‐Nearest Neighbors	0.70	0.75	0.75	0.75

## 3 Results and Discussion

**FIGURE 3:**
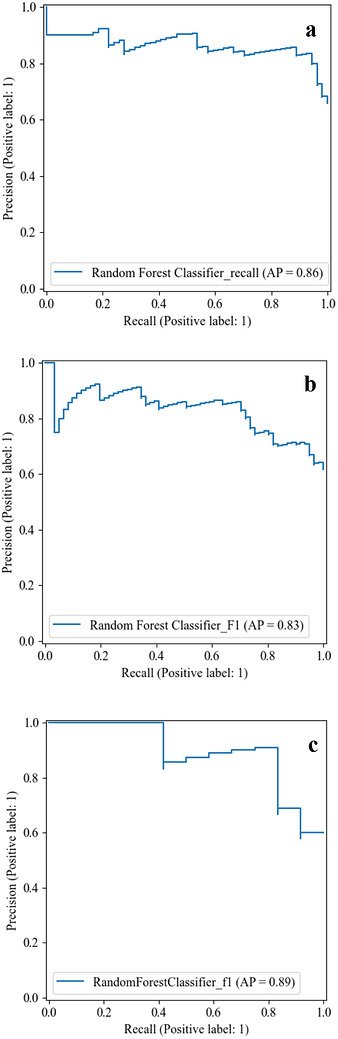
(a) Precision‐Recall (PR) curve of the Random Forest Classifier for the dataset of AMPs targeting *P. aeruginosa* PAO1, with recall as the optimization metric. The area under the curve (AUC) indicates an average precision (AP) of 0.86. (b) Precision‐Recall (PR) curve of the Random Forest Classifier for the dataset of AMPs targeting *S. aureus* ATCC 29213, with F1‐score as the optimization metric. The area under the curve (AUC) indicates an average precision (AP) of 0.83. (c) Precision‐Recall (PR) curve of the Random Forest Classifier for the dataset of AMPs targeting *M. tuberculosis* H37Rv and *M. smegmatis* mc^2^ 155 with F1‐score as the optimization metric. The area under the curve (AUC) indicates an average precision (AP) of 0.89.

For the dataset of AMPs targeting Gram‐negative *P. aeruginosa* PAO1, the different classifiers with optimal hyperparameters were obtained using the recall and F1‐score scoring metrics (Tables 1 and 2). The recall scoring‐metric‐based Random Forest model showed the highest F1‐score and precision compared to all other models and hyperparameters. The high recall of 0.87 means that 47 datapoints were identified as true positives compared to only 7 false negatives (in the test set) while a high precision of 0.85 means that 47 datapoints were identified as true positives compared to only 8 false positives, resulting in a high F1‐score of 0.86, and a robust accuracy of 0.82 further supports the selection of the Random Forest Classifier over other models. Although the Multi‐Layer Perception and C‐Support Vector hyperparameters using the recall scoring metric presented a perfect recall of 1.0, their F1‐scores were 0.79 and 0.84, respectively, and presented poor accuracies of 0.66 and 0.76; hence, they were deemed to be outcompeted by the Random Forest Classifier, which demonstrated a well‐balanced performance across multiple metrics. The average precision (AP) (See Figure 3a) was also analyzed to reaffirm the competence of the Random Forest model. The AP, which is calculated as the weighted mean of precision at different recall levels where the weight increases with recall was a high 0.86, further supporting the application of Random Forest Classifier hyperparameters obtained from the recall, scoring metric for SHAP analysis.

After following a similar methodology, for the AMP dataset targeting Gram‐positive bacteria *S. aureus* ATCC 29213 (Tables 3 and 4), the Random Forest Classifier with hyperparameters obtained from the F1‐score scoring metric showed the overall highest recall of 0.82, where 50 datapoints were true positives compared to 11 false negatives. The precision was also quite high, 0.75, from 50 true positives and 17 false positives, resulting in the overall highest F1‐score of 0.78. The overall accuracy was also the highest of 0.72, indicating a good balance across different performance metrics. The AP (See Figure 3b) was a high value of 0.83, additionally validating the appropriateness of using the Random Forest Classifier hyperparameters obtained from the F1‐score scoring‐metric for SHAP analysis of AMPs targeting *S. aureus* ATCC 29213.

For the AMP dataset targeting mycobacteria *M. tuberculosis* H37Rv and *M. smegmatis* mc^2^ 155 (Tables 5 and 6), the Random Forest Classifier with hyperparameters obtained from the F1‐score scoring metric showed a considerably high recall of 0.83 from 10 true positives compared to 2 false negatives and highest precision of 0.83 from 10 true positives and 2 false positives, resulting in the highest F1‐score of 0.83 and highest accuracy of 0.80 with a high AP value of 0.89 (See Figure 3c). Interestingly, whether the hyperparameters were obtained by the recall or F1‐score scoring metric, the best performing models for each dataset was the Random Forest Classifier and all had considerably high AP values exceeding 0.8, as well as their respective precision‐recall area under the curve (PR‐AUC), which is calculated using numerical integration, also exceeded 0.8 (See Figures S1–S3), justifying reliable SHAP analysis and straightforward comparisons of key peptide characteristics for significant antimicrobial activity against different microbe types.

**FIGURE 4:**
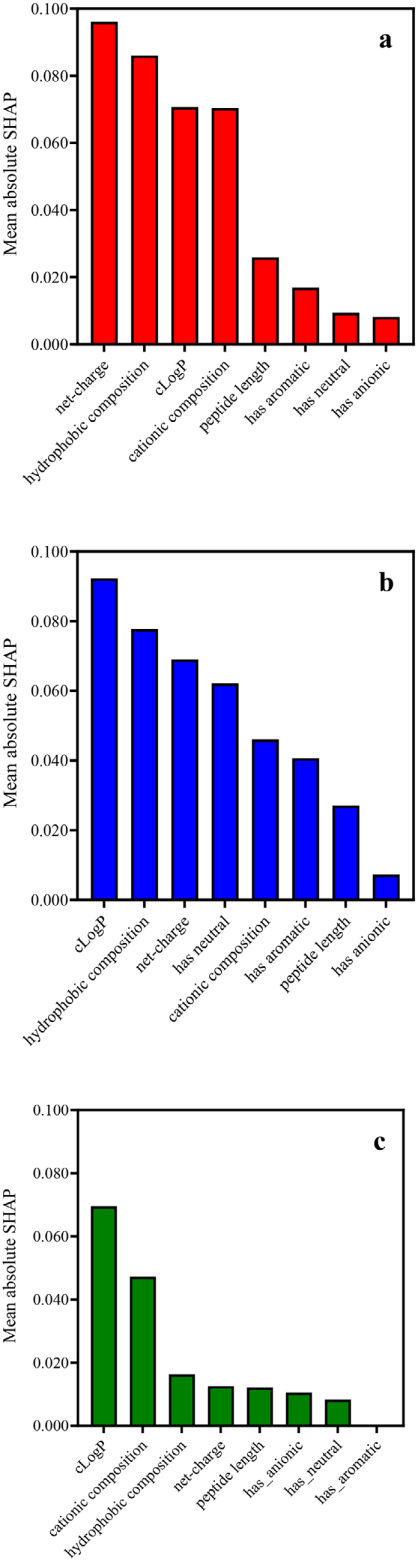
Bar plots showing the mean absolute SHAP value of features for AMPs targeting the four types of microbes, each obtained from the best performing hyperparameters of the Random Forest Classification model. Important features with a mean absolute SHAP collectively exceeding 80% of the total sum of the mean absolute SHAP values are ranked (in descending order of feature importance) as the following: (a) AMPs targeting *P. aeruginosa* PAO1 (red, hyperparameters obtained from the recall scoring‐metric): (1) net‐charge, (2) hydrophobic composition, (3) cLogP, and (4) cationic composition. (b) AMPs targeting *S. aureus* ATCC29213 (blue, hyperparameters obtained from the F1‐score scoring metric): (1) cLogP, (2) hydrophobic composition, (3) net‐charge, (4) the presence of neutral amino acids in their hydropathy in the peptide sequence, and (5) cationic composition. (c) AMPs targeting *M. tuberculosis* H37Rv and *M. smegmatis* mc^2^ 155 (green, hyperparameters obtained from the F1‐score scoring metric): (1) cLogP, (2) cationic composition, (3) hydrophobic composition. (4) net‐charge and (5) peptide length. The cLogP, net‐charge, cationic composition, and hydrophobic composition are important features for all three types of bacteria.

Global SHAP analyses of the three datasets were first conducted to identify the important features (See Figure 4). A feature was considered to be significant if the most impactful features collectively had mean absolute SHAP values that accounted for at least 80% of the total sum of mean SHAP absolute values. Other relatively less impactful features were considered to be unimportant. For AMPs targeting Gram‐negative bacterial strain *P. aeruginosa* PAO1, the important features (in descending order of importance) for predicting antibacterial efficacy were 1) net‐charge, 2) hydrophobic composition, 3) cLogP and 4) cationic composition. For the Gram‐positive bacterial strain *S. aureus* ATCC 29213, the important features were 1) cLogP, 2) hydrophobic composition, 3) net‐charge, 4) the presence of neutral amino acids in their hydropathy in the peptide sequence, and 5) cationic composition. For the mycobacterial strains of *M. tuberculosis* H37Rv and *M. smegmatis* mc^2^ 155 strains, the important features were 1) cLogP, 2) cationic composition, and 3) hydrophobic composition. 4) net‐charge and 5) peptide length.

**FIGURE 5:**
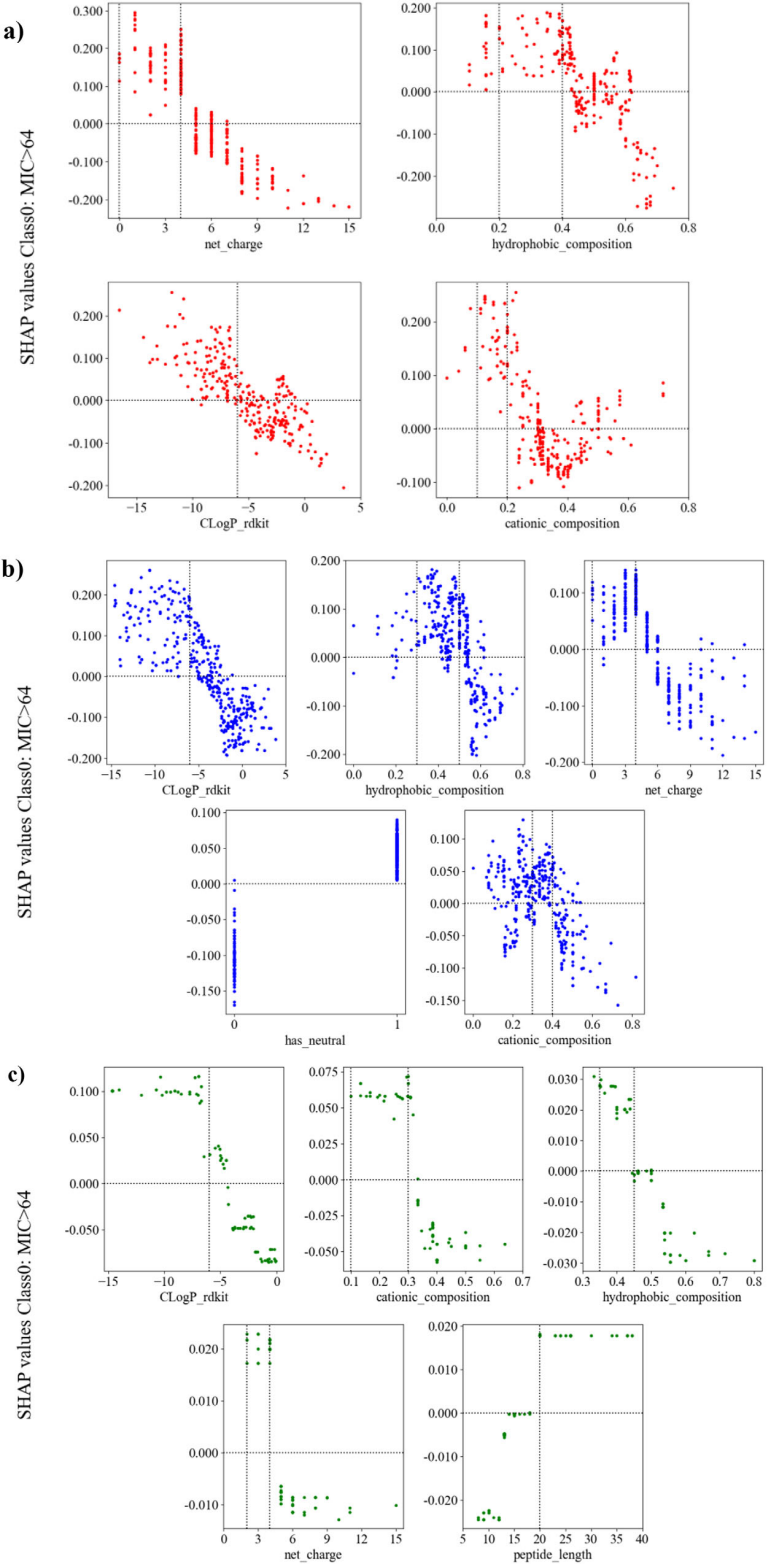
Local SHAP analysis in Class 0 for each target microbe. (a) The Random Forest Classification model with hyperparameters obtained from the recall scoring metric for four important features of AMPs targeting *P. aeruginosa* PAO1 (red) in descending order of importance from top‐left to bottom right. The horizontal dotted line indicates SHAP = 0, the vertical dotted lines indicate the ideal SHAP range or threshold for each feature. SHAP analysis suggests AMPs with a (in order of decreasing feature importance) net‐charge between 0 and +4, hydrophobic composition between 20%–40%, cLogP less than −6, and cationic composition between 10%–20% are predictive of antibacterial activity. (b) The Random Forest Classification model with hyperparameters obtained from the F1‐score scoring metric for five important features of AMPs targeting *S. aureus* ATCC 29213 (blue) in descending order of importance from top‐left to bottom right. The horizontal dotted line indicates SHAP = 0, the vertical dotted lines indicate the ideal SHAP range or threshold for each feature. SHAP analysis suggests AMPs with a (in order of decreasing feature importance from top left to bottom right) cLogP less than −6, hydrophobic composition 30%–50%, net‐charge 0 to +4, the presence of neutral (hydropathy‐wise) amino acids, and the cationic composition 30%–40% are predictive of antibacterial activity against *S. aureus* ATCC 29213. (c) The Random Forest Classification model with hyperparameters obtained from the F1‐score scoring metric for six important features of AMPs targeting *M. tuberculosis* H37Rv and *M. smegmatis* mc^2^ 155 (green) in descending order of importance from top left to bottom right. The horizontal dotted line indicates SHAP = 0, the vertical dotted lines indicate the ideal SHAP range or threshold for each feature. SHAP analysis suggests AMPs with a (in order of decreasing feature importance from top left to bottom right) cLogP less than −6, cationic composition of 10%–30%, hydrophobic composition of 35%–45%, net‐charge of +2 to +4, and the peptide length at least 20 to effectively target *M. tuberculosis* H37Rv and *M. smegmatis* mc^2^ 155.

Local SHAP analysis was conducted on the features deemed important to investigate their ideal thresholds or ranges (See Figure 5a–c). Interestingly, the required cLogP and net‐charge were the same for AMPs targeting all three types of bacteria, but there were small differences in the required hydrophobic and cationic compositions as well as the net‐charge of the AMPs targeting the three different types (See Table 7 for the recommended values/ranges). The identical cLogP threshold of ‐6 and net‐charge limit of +4 may suggest that the main antibacterial mechanism(s) between AMPs targeting the three types of bacteria are similar if not identical, where the folded secondary AMP structures sufficiently need to be hydrophilic as a prerequisite to actively fold into specific structures that can effectively diffuse through the peptidoglycan cell wall and actively take part in well‐distributed electrostatic interactions between the phospholipid head groups of the lipid bilayer which may result in pore or micelle formation or other nondestructive membrane altercations leading to cell death.

**TABLE 7:** Summary of the ideal ranges or thresholds (with positive SHAP values) of important features for AMPs targeting *P. aeruginosa* PAO1 Gram‐negative bacteria, *S. aureus* ATCC 29213 Gram‐positive bacteria, as well as *M. tuberculosis* H37Rv and *M. smegmatis* mc^2^ 155 mycobacteria. According to the SHAP analysis, the cLogP should be less than −6 for AMPs targeting all three types of bacteria, and the cLogP should be between +1.5 and +3.5. There a differences for the hydrophobic composition (10 20%–40% for AMPs targeting *P. aeruginosa* PAO1, 30%–50% for AMPs targeting *S. aureus* ATCC 29213, 35%–45% for AMPs targeting *M. tuberculosis* H37Rv and *M. smegmatis* mc^2^ 155, cationic composition (10%–20% for AMPs targeting *P. aeruginosa* PAO1, 30%–40% for AMPs targeting *S. aureus* ATCC 29213 and 10–30% for AMPs targeting *M. tuberculosis* H37Rv and *M. smegmatis* mc^2^ 155) and net‐charge (0 to +4 for AMPs targeting *P. aeruginosa* PAO1, 0 to +4 for *S. aureus* ATCC 29213 and +2 to +4 for AMPs targeting *M. tuberculosis* H37Rv and *M. smegmatis* mc^2^ 155). The inclusion of neutral amino acids in the peptide sequence is recommended for AMPs targeting *S. aureus* ATCC 29213. A peptide length of at least 20 for AMPs targeting *M. tuberculosis* H37Rv and *M. smegmatis* mc^2^ 155 is recommended.

	AMPs targeting Gram‐negative bacteria	AMPs targeting Gram‐positive bacteria	AMPs targeting mycobacteria
cLogP	Less than −6	Less than −6	Less than −6
Hydrophobic composition	20%–40%	30%–50%	35%–45%
Cationic composition	10%–20%	30%–40%	10%–30%
Net‐charge	0 to +4	0 to +4	+2 to +4
Neutral amino acids	—	Recommended	—
Peptide length	—	—	≥ 20

However, there were variations in the ideal cationic and hydrophobic composition for the AMPs targeting the three types of bacteria. The cationic and hydrophobic composition ranges for Gram‐positive *S. aureus* ATCC 29213 (cationic: 30%–40%, hydrophobic: 30%–50%) were higher compared to its Gram‐negative *P. aeruginosa* PAO1 counterpart (cationic: 10%–20%, hydrophobic: 20%–40%). For the AMPs targeting mycobacteria *M. tuberculosis* H37Rv and *M. smegmatis* mc^2^ 155 (cationic: 10%–30%, hydrophobic 35%–45%), the hydrophobic composition range was even higher than the other two bacterial types, yet the cationic composition range was relatively more moderate compared to the two. The compositional differences between the three types of bacteria can be attributed to their structural differences in the cell envelope.

A higher cationic composition is required for AMPs killing Gram‐positive bacteria and mycobacteria compared to the Gram‐negative bacteria counterpart, to effectively bind to the single inner membrane that is protected by cell walls that are relatively thicker than those of Gram‐negative bacteria. The ideal cationic composition range is higher for AMPs targeting Gram‐positive bacteria compared to AMPs targeting mycobacteria because of their substantially thicker cell wall, which requires a higher cationic composition for effective penetration. The higher hydrophobic component recommended to target Gram‐positive bacteria compared to Gram‐negative bacteria can also be attributed to the hydrophilic thick cell wall. AMPs with a low hydrophobicity may excessively interact with the cell wall and fail to reach the inner membrane. An even higher hydrophobic component recommended against mycobacteria is related to the highly hydrophobic mycolic layer, which may need to be actively disrupted prior to cell wall diffusion and eventual membrane disruption to induce cell death. Meanwhile, excessive hydrophobicity (e.g., exceeding 60%) even for Gram‐positive bacteria and mycobacteria may result in repulsion against the cell wall, and the AMP diffusion would also be unsuccessful, which may be the reason the recommended hydrophobic component for Gram‐positive bacteria is limited to 40% and for mycobacteria is limited to 45%. An excessively high cationic composition (e.g., exceeding 50%) and net‐charge (e.g., exceeding +5), where SHAP values of the AMPs for all three datasets are mostly negative, may result in aggregation and/or uneven distributions in electrostatic interactions between the AMPs and the bacterial membrane, resulting in antibacterial inactivity for all bacterial types. Also, excessive charge may result in the AMPs over‐interacting with the peptidoglycan cell wall which contains carboxyl groups, especially for the case of Gram‐positive bacteria, which consist of negative WTA and LTA teichoic acids, which may hinder their interaction with the inner‐membrane, which explains why the net‐charge limit is +4, even though the recommended cationic composition is higher than for AMPs targeting Gram‐negative bacteria and mycobacteria.

Interestingly, the inclusion of neutral amino acids in the peptide sequence is beneficial for AMPs targeting Gram‐positive bacteria. This may be the case because neutral amino acids have sufficient hydrophilicity to interact with the thick cell wall but are also hydrophobic enough to avoid being entrapped in the cell wall – aiding effective peptide diffusion. On the other hand, a peptide length of at least 20 is recommended for AMPs targeting mycobacteria. The long peptide length under the same peptide composition generally contributes to a lower charge‐to‐hydrophobicity ratio, which may further aid active disruption of the very hydrophobic mycolic acid layer, while avoiding an excessive hydrophobic composition which hinders cell wall penetration. These two qualitative features do not bear much significance for Gram‐negative bacteria, perhaps due to their much thinner cell wall and unique electronegative LPS‐rich OM.

In summary, this study, which employs RFC models for AMPs killing Gram‐negative bacteria, Gram‐positive bacteria, and mycobacteria, effectively aligns with previously proposed antibacterial mechanisms but also showcases the differences in the important criteria which directly relate to their structural differences. Similar ML techniques could potentially be employed in the future to investigate in depth how the criteria differ based on the microbial species and its strain of the same type of microbe to effectively select or develop an AMP to target a microbial strain of specific interest.

## 4 Conclusions

AMPs exhibit distinct antibacterial efficacies against different bacterial species and strains depending on their unique peptide sequences. To streamline the selection of effective peptides, Random Forest Classification ML models were applied to datasets of AMPs targeting Gram‐negative bacterial strain *P. aeruginosa* PAO1, Gram‐positive bacterial strain *S. aureus* ATCC 29213 and, mycobacterial strains *M. tuberculosis* H37Rv and *M. smegmatis* mc^2^ 155, where an AMP was assumed to be effective if it presented a MIC_90_ of 64 µg mL^−1^ or less to investigate their important features and ideal ranges and thresholds for predicting antibacterial efficacy. The Random Forest Classification models suggest that the cLogP of the peptides should be less than −6 and net‐charge should be limited to +4 for all three types of bacteria. In the case of the hydrophobic composition, 20%–40% for Gram‐negative bacteria, 30%–50% for Gram‐positive bacteria, and 35%–45% for mycobacteria were recommended for significant antibacterial activity. The hydrophobic composition positively correlated with the hydrophobicity of the bacterial type. In the case of the cationic composition, 10%–20% for Gram‐negative bacteria, 30%–40% for Gram‐positive bacteria, and 10%–30% for mycobacteria were recommended. The cationic composition positively correlated with the cell wall thickness of the bacterial type. In addition, the inclusion of amino acids considered neutral in their hydropathy were recommended for targeting Gram‐positive bacteria, perhaps to aid thick cell wall diffusion, and a peptide length of at least 20 was recommended for targeting mycobacteria, perhaps to aid active mycolic acid disruption. Although the unique amino acid order of an individual peptide determines its specific antimicrobial efficacy, peptides that fulfill the broader key characteristics apart from its unique amino acid order may be significantly more effective than other peptide sequences overall, for each microbe target of interest – aiding the selection or discovery of high‐performing AMPs.

## Conflicts of Interest

The authors declare no conflicts of interest.

## Supporting information




**Supporting File**: marc70069‐sup‐0001‐SuppMat.docx.

## Data Availability

The data that support the findings of this study are available from the corresponding author upon reasonable request.

## References

[1] H. Hanberger , M. Antonelli , M. Holmbom , et al., “Infections, Antibiotic Treatment and Mortality in Patients Admitted to ICUs in Countries Considered to Have High Levels of Antibiotic Resistance Compared to those With Low Levels,” BMC Infectious Diseases 14 (2014): 513. 25245620 10.1186/1471-2334-14-513 PMC4181425

[2] G. D. Wright , “Solving the Antibiotic Crisis,” ACS Infectious Diseases 1 (2015): 80–84. 27622298 10.1021/id500052s

[3] J. H. Rex , B. I. Eisenstein , J. Alder , et al., “A Comprehensive Regulatory Framework to Address the Unmet Need for New Antibacterial Treatments,” The Lancet Infectious Diseases 13 (2013): 269–275. 23332713 10.1016/S1473-3099(12)70293-1

[4] F. Rong , Y. Tang , T. Wang , et al., “Nitric Oxide‐Releasing Polymeric Materials for Antimicrobial Applications: A Review,” Antioxidants 8 (2019): 556. 31731704 10.3390/antiox8110556 PMC6912614

[5] P. Li , X. Li , R. Saravanan , C. M. Li , and S. Su , “Antimicrobial Macromolecules: Synthesis Methods and Future Applications,” RSC Advances 2 (2012): 4031–4044.

[6] O. Dramé , D. Leclair , E. J. Parmley , et al., “Antimicrobial Resistance of Campylobacter in Broiler Chicken Along the Food Chain in Canada,” Foodborne Pathogens and Disease 17 (2020): 512–520. 32130036 10.1089/fpd.2019.2752 PMC7415884

[7] World Health Organization . New Report Calls for Urgent Action to Avert Antimicrobial Resistance Crisis, WHO:, Geneva, 2019. (accessed May 24, 2025), Available online: https://www.who.int/news/item/29‐04‐2019‐new‐report‐calls‐for‐urgent‐action‐to‐avert‐antimicrobial‐resistance‐crisis.

[8] A. Harms , E. Maisonneuve , and K. Gerdes , “Mechanisms of Bacterial Persistence During Stress and Antibiotic Exposure,” Science 354 (2016): aaf4268. 27980159 10.1126/science.aaf4268

[9] L. Zhang and R. L. Gallo , “Antimicrobial Peptides,” Current Biology 26 (2016): R14–R19. 26766224 10.1016/j.cub.2015.11.017

[10] E. F. Haney , S. C. Mansour , and R. E. W. Hancock , “Antimicrobial Peptides: an Introduction,” Methods in Molecular Biology 1548 (2017): 3–22. 28013493 10.1007/978-1-4939-6737-7_1

[11] Z. Y. Ong , N. Wiradharma , and Y. Y. Yang , “Strategies Employed in the Design and Optimization of Synthetic Antimicrobial Peptide Amphiphiles With Enhanced Therapeutic Potentials,” Advanced Drug Delivery Reviews 78 (2014): 28–45. 25453271 10.1016/j.addr.2014.10.013

[12] R. E. W. Hancock and H.‐G. Sahl , “Antimicrobial and Host‐Defense Peptides as New Anti‐Infective Therapeutic Strategies,” Nature Biotechnology 24 (2006): 1551–1557. 10.1038/nbt1267 17160061

[13] M. Zasloff , “Antimicrobial Peptides of Multicellular Organisms,” Nature 415 (2002): 389–395. 11807545 10.1038/415389a

[14] Z. Jiang , A. I. Vasil , J. Hale , R. E. W. Hancock , M. L. Vasil , and R. S. Hodges , “Effects of Net Charge and the Number of Positively Charged Residues on the Biological Activity of Amphipathic Alpha‐Helical Cationic Antimicrobial Peptides,” Advances in Experimental Medicine and Biology 611 (2009): 561–562. 19400313 10.1007/978-0-387-73657-0_246

[15] A. Moretta , R. Salvia , C. Scieuzo , et al., “A Bioinformatic Study of Antimicrobial Peptides Identified in the Black Soldier Fly (BSF) Hermetia Illucens (Diptera: Stratiomyidae),” Scientific Reports 10 (2020): 16875. 33037295 10.1038/s41598-020-74017-9 PMC7547115

[16] P. Bhadra , J. Yan , J. Li , S. Fong , and S. W. I. Siu , “AmPEP: Sequence‐Based Prediction of Antimicrobial Peptides using Distribution Patterns of Amino Acid Properties and Random Forest,” Scientific Reports 8 (2018): 1697. 29374199 10.1038/s41598-018-19752-w PMC5785966

[17] M. N. Melo , R. Ferre , and M. A. R. B. Castanho , “Antimicrobial Peptides: Linking Partition, Activity and High Membrane‐Bound Concentrations,” Nature Reviews Microbiology 7 (2009): 245–250. 19219054 10.1038/nrmicro2095

[18] G. Ehrenstein and H. Lecar , “Electrically Gated Ionic Channels in Lipid Bilayers,” Quarterly Reviews of Biophysics 10 (1977): 1. 327501 10.1017/s0033583500000123

[19] Y. Pouny , D. Rapaport , A. Mor , P. Nicolas , and Y. Shai , “Interaction of Antimicrobial Dermaseptin and Its Fluorescently Labeled Analogs With Phospholipid Membranes,” Biochemistry 31 (1992): 12416–12423. 1463728 10.1021/bi00164a017

[20] S. J. Ludtke , K. He , W. T. Heller , T. A. Harroun , L. Yang , and H. W. Huang , “Membrane Pores Induced by Magainin,” Biochemistry 35 (1996): 13723–13728. 8901513 10.1021/bi9620621

[21] F.‐Y. Chen , M.‐T. Lee , and H. W. Huang , “Evidence for Membrane Thinning Effect as the Mechanism for Peptide‐Induced Pore Formation,” Biophysical Journal 84 (2003): 3751–3758. 12770881 10.1016/S0006-3495(03)75103-0 PMC1302957

[22] S. Ludtke , K. He , and H. Huang , “Membrane Thinning Caused by Magainin 2,” Biochemistry 34 (1995): 16764–16769. 8527451 10.1021/bi00051a026

[23] G. Pabst , S. L. Grage , S. Danner‐Pongratz , et al., “Membrane Thickening by the Antimicrobial Peptide PGLa,” Biophysical Journal 95 (2009): 5779–5788. 10.1529/biophysj.108.141630 PMC2599817 18835902

[24] A. Patrzykat , C. L. Friedrich , L. Zhang , V. Mendoza , and R. E. Hancock , “Sublethal Concentrations of Pleurocidin‐Derived Antimicrobial Peptides Inhibit Macromolecular Synthesis in Escherichia Coli,” Antimicrobial Agents and Chemotherapy 46 (2002): 605–614. 11850238 10.1128/AAC.46.03.605-614.2002 PMC127508

[25] C. B. Park , H. S. Kim , and S. C. Kim , “Mechanism of Action of the Antimicrobial Peptide Buforin II: Buforin II Kills Microorganisms by Penetrating the Cell Membrane and Inhibiting Cellular Functions,” Biochemical and Biophysical Research Communications 244 (1998): 253–257. 9514864 10.1006/bbrc.1998.8159

[26] C. F. Le , C. M. Fang , and S. D. Sekaran , “Intracellular Targeting Mechanisms by Antimicrobial Peptides,” Antimicrobial Agents and Chemotherapy 61 (2017): 02340. 10.1128/AAC.02340-16 PMC5365711 28167546

[27] P. Muthirulan and A. R. Chandrasekaran , “Microbial Flow Cytometry: an Ideal Tool for Prospective Antimicrobial Drug Development,” Analytical Biochemistry 509 (2016): 89–91. 27288557 10.1016/j.ab.2016.05.025

[28] L. Yu , K. Li , J. Zhang , et al., “Antimicrobial Peptides and Macromolecules for Combating Microbial Infections: From Agents to Interfaces,” ACS Applied Bio Materials 5 (2022): 366–393. 10.1021/acsabm.1c01132 35072444

[29] N. Wiradharma , M. Y. S. Sng , M. Khan , Z.‐Y. Ong , and Y.‐Y. Yang , “Rationally Designed α‐Helical Broad‐Spectrum Antimicrobial Peptides With Idealized Facial Amphiphilicity,” Macromolecular Rapid Communications 34 (2012): 74–80. 23112127 10.1002/marc.201200534

[30] P. S. Bisen , M. Debnath , and S. Prasad , “Identification and Classification of Microbes,” Microbes: Concepts and Applications, 275–337, (Wiley‐Blackwell, 2012).

[31] A. C. Engler , N. Wiradharma , Z. Y. Ong , D. J. Coady , J. L. Hedrick , and Y.‐Y. Yang , “Emerging Trends in Macromolecular Antimicrobials to Fight Multi‐Drug‐Resistant Infections,” Nano Today 7 (2012): 201–222.

[32] M.‐P. Mingeot‐Leclercq and J.‐L. Décout , “Bacterial Lipid Membranes as Promising Targets to Fight Antimicrobial Resistance, Molecular Foundations and Illustration Through the Renewal of Aminoglycoside Antibiotics and Emergence of Amphiphilic Aminoglycosides,” MedChemComm 7 (2016): 586–611.

[33] A. H. Takade , A. Umeda , M. Matsuoka , S. Yoshida , M. Nakamura , and K. Amako , “Comparative Studies of the Cell Structures of Mycobacterium leprae and M. tuberculosis Using the Electron Microscopy Freeze‐Substitution Technique,” Microbiology and Immunology 47 (2003): 265–270. 12801063 10.1111/j.1348-0421.2003.tb03394.x

[34] L. J. Alderwick , J. Harrison , G. S. Lloyd , and H. L. Birch , “The Mycobacterial Cell Wall—Peptidoglycan and Arabinogalactan,” Cold Spring Harbor Perspectives in Medicine 5 (2015): a021113. 25818664 10.1101/cshperspect.a021113 PMC4526729

[35] H. J. Marrakchi , M.‐A. Lanéelle , and M. Daffé , “Mycolic Acids: Structures, Biosynthesis, and Beyond,” Chemistry & Biology 21 (2014): 67–85. 24374164 10.1016/j.chembiol.2013.11.011

[36] V. K. Jarlier and H. Nikaido , “Mycobacterial Cell Wall: Structure and Role in Natural Resistance to Antibiotics,” FEMS Microbiology Letters 123 (1994): 11–18. 7988876 10.1111/j.1574-6968.1994.tb07194.x

[37] A. Shahraki , M. Abbasi , and Ø. Haugen , “Boosting Algorithms for Network Intrusion Detection: a Comparative Evaluation of Real AdaBoost, Gentle AdaBoost and Modest AdaBoost,” Engineering Applications of Artificial Intelligence 94 (2020): 103770.

[38] S. S. Hussain and S. S. H. Zaidi , “AdaBoost Ensemble Approach With Weak Classifiers for Gear Fault Diagnosis and Prognosis in DC Motors,” Applied Sciences 14 (2024): 3105.

[39] O. Kramer Dimensionality Reduction with Unsupervised Nearest Neighbors, (Springer, 2013).

[40] H. Drucker , L. Kaufman , and V. Vapnik , “Support Vector Regression Machines,” Advances in Neural Information Processing Systems 28 (1997): 779–784.

[41] R. Kumar , B. Goswami , S. M. Mhatre , and S. Agrawal , “Naive Bayes in Focus: a Thorough Examination of Its Algorithmic Foundations and Use Cases,” International Journal of Innovative Science and Research Technology (IJISRT) 9 (2024): 2078–2081.

[42] E. Bilgi and C. O. Karakus , “Machine Learning‐Assisted Prediction of the Toxicity of Silver Nanoparticles: a Meta‐Analysis,” Journal of Nanoparticle Research 25 (2023): 157.

[43] W. Ge , R. De Silva , Y. Fan , S. A. Sisson , and M. H. Stenzel , “Machine Learning in Polymer Research,” Advanced Materials 37 (2025): 2413695. 39924835 10.1002/adma.202413695 PMC11923530

[44] J. S. Cobb , M. A. Seale , and A. V. Janorkar , “Evaluation of Machine Learning Algorithms to Predict the Hydrodynamic Radii and Transition Temperatures of Chemo‐Biologically Synthesized Copolymers,” Computers in Biology and Medicine 128 (2021): 104134. 33249343 10.1016/j.compbiomed.2020.104134 PMC7775344

[45] Y. Y. Song and L. U. Ying , “Decision Tree Methods: Applications for Classification and Prediction,” Shanghai Archives of Psychiatry 27 (2015): 130–135. 26120265 10.11919/j.issn.1002-0829.215044 PMC4466856

[46] J. P. Lightstone , L. Chen , C. Kim , R. Batra , and R. Ramprasad , “Refractive Index Prediction Models for Polymers Using Machine Learning,” Journal of Applied Physics 127 (2020): 213913.

[47] L. Breiman , “Random Forests,” Machine Learning 45 (2001): 5–32.

[48] L. Tao , V. Varshney , and Y. Li , “Benchmarking Machine Learning Models for Polymer Informatics: an Example of Glass Transition Temperature,” Journal of Chemical Information and Modeling 61 (2021): 5395–5413. 34662106 10.1021/acs.jcim.1c01031

[49] A. Tiihonen , S. J. Cox‐Vazquez , Q. Liang , et al., “Predicting Antimicrobial Activity of Conjugated Oligoelectrolyte Molecules via Machine Learning,” Journal of the American Chemical Society 143 (2021): 18917–18931. 34739239 10.1021/jacs.1c05055

[50] V. Kundi , Y. Jin , A. Chandrasekaran , et al., “Machine Learning Prediction of Antibacterial Activity of Block Copolymers,” ACS Applied Nano Materials 7 (2024): 8939–8948.

[51] P. T. Phuong , S. Oliver , J. He , E. H. H. Wong , R. T. Mathers , and C. Boyer , “Effect of Hydrophobic Groups on Antimicrobial and Hemolytic Activity: Developing a Predictive Tool for Ternary Antimicrobial Polymers,” Biomacromolecules 21 (2020): 5241–5255. 33186496 10.1021/acs.biomac.0c01320

[52] P. R. Judzewitsch , L. Zhao , S. Edgar , and C. Boyer , “High‐Throughput Synthesis of Antimicrobial Copolymers and Rapid Evaluation of Their Bioactivity,” Macromolecules 52 (2019): 3975–3986.

[53] P. R. Judzewitsch , T. Nguyen , S. Shanmugam , E. H. H. Wong , and C. Boyer , “Towards Sequence‐Controlled Antimicrobial Polymers: Effect of Polymer Block Order on Antimicrobial Activity,” Angewandte Chemie International Edition 57 (2018): 4559–4564. 29441657 10.1002/anie.201713036

[54] P. R. Judzewitsch , N. Corrigan , F. Trujillo , et al., “High‐Throughput Process for the Discovery of Antimicrobial Polymers and Their Upscaled Production via Flow Polymerization,” Macromolecules 53 (2020): 631–639.

[55] Dbaasp.org. (accessed May 11, 2025), https://www.dbaasp.org/search.

[56] J. Kyte and R. F. Doolittle , “A Simple Method for Displaying the Hydropathic Character of a Protein,” Journal of Molecular Biology 157 (1982): 105–132. 7108955 10.1016/0022-2836(82)90515-0

[57] W. McKinney , “Data Structures for Statistical Computing in Python,” Proceeding 9th Python Science Conference (2010): 56–61.

[58] The Pandas Development Team . pandas‐dev/pandas: *Pandas* , Zenodo, 2020. (accessed May 21, 2025), 10.5281/zenodo.3509134 .

[59] C. R. Harris , K. J. Millman , S. J. van der Walt , et al., “Array Programming With NumPy,” Nature 585 (2020): 357–362. 32939066 10.1038/s41586-020-2649-2 PMC7759461

[60] F. Pedregosa , “Scikit‐Learn: Machine Learning in Python,” Journal of Machine Learning Research 12 (2011): 2825–2830.

[61] N. Brown , “Appendix D: RDKit. In Silico Medicinal Chemistry: Computational Methods to Support Drug Design,” Royal Society of Chemistry, (Cambridge, 2015), 199–200.

[62] S. M. Lundberg and S.‐I. Lee , Advances in Neural Information Processing Systems 30, eds. I. Guyon , U.V. Luxburg , S. Bengio , et al., (Curran Associates, Inc., 2017), 4765–4774.

