# Supplementary material for: Uncovering Key Characteristics of Antibacterial Peptides through Machine Learning
Source: Macromol Rapid Commun. 2025 Sep 28;47(12):e00583. doi: 10.1002/marc.202500583 (PMC13309147; doi:10.1002/marc.202500583)
Supplement: Supplementary file 1 — Supporting File: marc70069‐sup‐0001‐SuppMat.docx. [file MARC-47-e00583-s001.docx]

Supporting Information

Uncovering Key Characteristics of Antibacterial Peptides Through Machine Learning

Jooyoung Roh^1^, Cyrille Boyer^1,2^ and Priyank Kumar^1^

1. School of Chemical Engineering, University of New South Wales (UNSW), Sydney, New South Wales 2052, Australia

2. Australian Centre for NanoMedicine, UNSW, Sydney, New South Wales 2052, Australia

*Corresponding author: [cboyer@unsw.edu.au](mailto:cboyer@unsw.edu.au); priyank.kumar@unsw.edu.au

Data Availability

The codes used in the study can be found in the following link: <https://github.com/rohjooyoung/AMPs_gram_negative_vs_gram_positive>

**Table S1**: Optimized hyperparameters for all the classification models using both ‘recall’ and ‘F1’ the scoring metrics of AMPs targeting *Pseudomonas aeruginosa* PAO1.

| Classification Model | Scoring Metric | Optimized Hyperparameters |
| --- | --- | --- |
| Decision Tree | Recall | DecisionTreeClassifier(min_samples_leaf=3, max_features='sqrt', max_depth=7, criterion='log_loss', class_weight='balanced',  random_state=0, splitter='best') |
|  | F1 | DecisionTreeClassifier(min_samples_leaf=2, max_features='sqrt', max_depth=29, criterion='log_loss', class_weight='balanced', random_state=0, splitter='best') |
| Random Forest | Recall | RandomForestClassifier(n_estimators=352, min_samples_leaf=2, min_samples_split=3, max_depth=24, max_features='log2', criterion='entropy', class_weight='balanced_subsample',   random_state=0, bootstrap=True) |
|  | F1 | RandomForestClassifier( n_estimators=83, min_samples_leaf=2, min_samples_split=5, max_depth=17, max_features='log2', criterion='log_loss', class_weight='balanced_subsample', random_state=0, bootstrap=True) |
| Multi-Layer Perception | Recall | MLPClassifier(hidden_layer_sizes=124, learning_rate='constant', max_iter=460, alpha=0.095, solver='sgd', activation='logistic', random_state=0) |
|  | F1 | MLPClassifier(hidden_layer_sizes=96, learning_rate='adaptive', max_iter=327, alpha=0.054, solver='lbfgs', activation='tanh', random_state=0) |
| Logistic Regression | Recall | LogisticRegression(C=38.491, max_iter=408, class_weight='balanced', random_state=0) |
|  | F1 | LogisticRegression(C=17.073, max_iter=372, class_weight='balanced', random_state=0) |
| Gaussian Naïve Bayes | Recall | GaussianNB(var_smoothing=0.000466) |
|  | F1 | GaussianNB(var_smoothing=0.0536) |
| C-Support Vector | Recall | SVC(kernel='rbf', C=42.912, gamma=0.935, class_weight='balanced', random_state=0, probability=True) |
|  | F1 | SVC(kernel='poly', C=5.387, gamma=0.862, class_weight='balanced', random_state=0, probability=True) |
| k-Nearest Neighbour | Recall | KNeighborsClassifier(n_neighbors=49, leaf_size=43, p=1, algorithm='ball_tree', weights='uniform') |
|  | F1 | KNeighborsClassifier(n_neighbors=11, leaf_size=3, p=2, algorithm='ball_tree', weights='distance') |

**Table S2**: Optimized hyperparameters for seven classification models using both ‘recall’ and ‘F1’ the scoring metrics of the AMPs targeting *Staphylococcus aureus* ATCC 29213.

| Classification Model | Scoring Metric | Optimized Hyperparameters |
| --- | --- | --- |
| Decision Tree | Recall | DecisionTreeClassifier(min_samples_leaf=3, max_features='sqrt', max_depth=13, criterion='entropy', class_weight='balanced', random_state=0, splitter='best') |
|  | F1 | DecisionTreeClassifier( min_samples_leaf=4, max_features='sqrt', max_depth=14, criterion='entropy', class_weight='balanced', random_state=0, splitter='best') |
| Random Forest | Recall | RandomForestClassifier( n_estimators=311, min_samples_leaf=2, min_samples_split=4, max_depth=19, max_features='log2', criterion='log_loss', class_weight='balanced', random_state=0, bootstrap=True) |
|  | F1 | RandomForestClassifier(n_estimators=300, min_samples_leaf=2, min_samples_split=4, max_depth=15, max_features='log2', criterion='entropy', class_weight='balanced', random_state=0, bootstrap=True) |
| Multi-Layer Perception | Recall | MLPClassifier(hidden_layer_sizes=20, learning_rate='adaptive', max_iter=240, alpha=0.0855, solver='sgd', activation='logistic', random_state=0) |
|  | F1 | MLPClassifier(hidden_layer_sizes=147, learning_rate='constant', max_iter=371, alpha=0.0891, solver='adam', activation='tanh', random_state=0) |
| Logistic Regression | Recall | LogisticRegression(C=23.116, max_iter=27, class_weight='balanced', random_state=0) |
|  | F1 | LogisticRegression( C=55.464, max_iter=9, class_weight='balanced', random_state=0) |
| Gaussian Naïve Bayes | Recall | GaussianNB(var_smoothing=0.000334) |
|  | F1 | GaussianNB(var_smoothing=0.000345) |
| C-Support Vector | Recall | SVC( kernel='rbf', C=33.955, gamma=0.0229, class_weight='balanced', random_state=0, probability=True) |
|  | F1 | SVC(kernel='rbf', C=39.910, gamma=0.0284, class_weight='balanced', random_state=0, probability=True) |
| k-Nearest Neighbour | Recall | KNeighborsClassifier(n_neighbors=47, leaf_size=47, p=1, algorithm='kd_tree', weights='distance') |
|  | F1 | KNeighborsClassifier(n_neighbors=9, leaf_size=41, p=2, algorithm='auto', weights='uniform') |

**Table S3**: Optimized hyperparameters for seven classification models using both ‘recall’ and ‘F1’ the scoring metrics of the AMPs targeting *Mycobacterium tuberculosis* H37Rv and *Mycobacterium smegmatis* mc2 155.

| Classification Model | Scoring Metric | Optimized Hyperparameters |
| --- | --- | --- |
| Decision Tree | Recall | DecisionTreeClassifier(random_state=0, min_samples_leaf=3, max_features='log2', max_depth=27, criterion='entropy', class_weight='balanced', splitter='best') |
|  | F1 | DecisionTreeClassifier(random_state=0, min_samples_leaf=2, max_features='log2', max_depth=24, criterion='gini', class_weight='balanced', splitter='best') |
| Random Forest | Recall | RandomForestClassifier(class_weight='balanced', criterion='entropy', max_depth=10, max_features='sqrt', min_samples_leaf=2, min_samples_split=5, n_estimators=57, bootstrap=True, random_state=0) |
|  | F1 | RandomForestClassifier(class_weight='balanced', criterion='entropy', max_depth=19, max_features='sqrt', min_samples_leaf=9, min_samples_split=6, n_estimators=29, bootstrap=True, random_state=0) |
| Multi-Layer Perception | Recall | MLPClassifier(activation='logistic', alpha=0.055235137906738625, hidden_layer_sizes=112, learning_rate='adaptive', max_iter=437, random_state=0, solver='sgd') |
|  | F1 | MLPClassifier(activation='logistic', alpha=0.08243775083360846, hidden_layer_sizes=60, learning_rate='constant', max_iter=271, random_state=0, solver='lbfgs') |
| Logistic Regression | Recall | LogisticRegression(random_state=0, max_iter=88, class_weight='balanced', C=22.896007244407233) |
|  | F1 | LogisticRegression(random_state=0, max_iter=272, class_weight='balanced', C=0.33583842323318946) |
| Gaussian Naïve Bayes | Recall | GaussianNB(var_smoothing=0.0009633176661125813) |
|  | F1 | GaussianNB(var_smoothing=0.00015204739205871616) |
| C-Support Vector | Recall | SVC(kernel='rbf', gamma=0.7587050262057607, class_weight='balanced', C=71.37256691715945, random_state=0, probability=True) |
|  | F1 | SVC(kernel='poly', gamma=0.051062638693503484, class_weight='balanced', C=22.47608666946083, random_state=0, probability=True) |
| k-Nearest Neighbour | Recall | KNeighborsClassifier(weights='distance', p=1, n_neighbors=4, leaf_size=34, algorithm='auto') |
|  | F1 | KNeighborsClassifier(weights='distance', p=2, n_neighbors=3, leaf_size=5, algorithm='auto') |

**Table S4**: Summary of all the Classification Models When Scoring is Set to Recall for AMPs targeting [*P. aeruginosa* PAO1](https://www.bing.com/ck/a?!&&p=f230aa10195c9d06f193d941708ba1a1dcbe286ac86d39ce7407ca47ecb68d3bJmltdHM9MTc0NTAyMDgwMA&ptn=3&ver=2&hsh=4&fclid=082e62e2-70d8-6464-3b6e-712f71d7658a&psq=PAO1&u=a1aHR0cHM6Ly93d3cubmNiaS5ubG0ubmloLmdvdi9udWNjb3JlL05DXzAwMjUxNg&ntb=1) of the “train” set. The Random Forest Classifier, highlighted in bold, is chosen as the best-performing model given its well-balanced performance across different metrics based on its performance on the “test” set (**See Table 1**).

| Models | Accuracy | F1 | Recall | Precision |
| --- | --- | --- | --- | --- |
| **Random Forest** | **0.96** | **0.97** | **0.96** | **0.98** |
| Decision Tree | 0.80 | 0.85 | 0.83 | 0.86 |
| Multi-Layer Perception | 0.66 | 0.80 | 1.0 | 0.66 |
| C-Support Vector | 0.98 | 0.98 | 0.97 | 1.0 |
| Logistic Regression | 0.71 | 0.76 | 0.68 | 0.86 |
| Naïve Bayes | 0.69 | 0.75 | 0.72 | 0.79 |
| kNN | 0.69 | 0.80 | 0.93 | 0.70 |

| Models | Accuracy | F1 | Recall | Precision |
| --- | --- | --- | --- | --- |
| Random Forest | 0.96 | 0.97 | 0.96 | 0.98 |
| Decision Tree | 0.86 | 0.89 | 0.83 | 0.96 |
| Multi-Layer Perception | 0.98 | 0.98 | 0.99 | 0.98 |
| C-Support Vector | 0.95 | 0.96 | 0.94 | 0.99 |
| Logistic Regression | 0.71 | 0.76 | 0.68 | 0.86 |
| Naïve Bayes | 0.69 | 0.76 | 0.73 | 0.79 |
| kNN | 0.99 | 0.99 | 0.99 | 1.0 |

**Table S5**: Summary of all the Classification Models When Scoring is Set to F1-score for AMPs targeting [*P. aeruginosa* PAO1](https://www.bing.com/ck/a?!&&p=f230aa10195c9d06f193d941708ba1a1dcbe286ac86d39ce7407ca47ecb68d3bJmltdHM9MTc0NTAyMDgwMA&ptn=3&ver=2&hsh=4&fclid=082e62e2-70d8-6464-3b6e-712f71d7658a&psq=PAO1&u=a1aHR0cHM6Ly93d3cubmNiaS5ubG0ubmloLmdvdi9udWNjb3JlL05DXzAwMjUxNg&ntb=1) of the “train” set.

**Table S6**: Summary of all the Classification Models When Scoring is Set to Recall for AMPs targeting [*S. aureus* ATCC](https://www.bing.com/ck/a?!&&p=f230aa10195c9d06f193d941708ba1a1dcbe286ac86d39ce7407ca47ecb68d3bJmltdHM9MTc0NTAyMDgwMA&ptn=3&ver=2&hsh=4&fclid=082e62e2-70d8-6464-3b6e-712f71d7658a&psq=PAO1&u=a1aHR0cHM6Ly93d3cubmNiaS5ubG0ubmloLmdvdi9udWNjb3JlL05DXzAwMjUxNg&ntb=1) 29213 of the “train” set.

| Models | Accuracy | F1 | Recall | Precision |
| --- | --- | --- | --- | --- |
| Random Forest | 0.98 | 0.99 | 0.99 | 0.98 |
| Decision Tree | 0.78 | 0.82 | 0.79 | 0.85 |
| Multi-Layer Perception | 0.62 | 0.77 | 1.0 | 0.62 |
| C-Support Vector | 0.82 | 0.86 | 0.85 | 0.86 |
| Logistic Regression | 0.74 | 0.77 | 0.72 | 0.84 |
| Naïve Bayes | 0.72 | 0.76 | 0.73 | 0.80 |
| kNN | 1.0 | 1.0 | 1.0 | 1.0 |

**Table S7**: Summary of all the Classification Models When Scoring is Set to F1-score for AMPs targeting [*S. aureus* ATCC](https://www.bing.com/ck/a?!&&p=f230aa10195c9d06f193d941708ba1a1dcbe286ac86d39ce7407ca47ecb68d3bJmltdHM9MTc0NTAyMDgwMA&ptn=3&ver=2&hsh=4&fclid=082e62e2-70d8-6464-3b6e-712f71d7658a&psq=PAO1&u=a1aHR0cHM6Ly93d3cubmNiaS5ubG0ubmloLmdvdi9udWNjb3JlL05DXzAwMjUxNg&ntb=1) 29213 of the “train” set. The Random Forest Classifier, highlighted in bold, is chosen as the best-performing model given its well-balanced performance across different metrics based on its performance on the “test” set (**See Table 4**).

| Models | Accuracy | F1 | Recall | Precision |
| --- | --- | --- | --- | --- |
| **Random Forest** | **0.98** | **0.99** | **0.99** | **0.98** |
| Decision Tree | 0.85 | 0.88 | 0.83 | 0.93 |
| Multi-Layer Perception | 0.82 | 0.86 | 0.90 | 0.83 |
| C-Support Vector | 0.84 | 0.87 | 0.86 | 0.88 |
| Logistic Regression | 0.74 | 0.78 | 0.72 | 0.84 |
| Naïve Bayes | 0.72 | 0.76 | 0.73 | 0.80 |
| kNN | 0.79 | 0.83 | 0.84 | 082 |

**Table S8**: Summary of all the Classification Models When Scoring is Set to Recall for AMPs targeting *M. tuberculosis* H37Rv and *M. smegmatis* mc2 155 of the “train” set.

| Models | Accuracy | F1 | Recall | Precision |
| --- | --- | --- | --- | --- |
| Random Forest | 0.92 | 0.93 | 0.92 | 0.94 |
| Decision Tree | 0.88 | 0.90 | 0.89 | 0.92 |
| Multi-Layer Perception | 0.62 | 0.76 | 1.0 | 0.62 |
| C-Support Vector | 0.92 | 0.94 | 0.97 | 0.90 |
| Logistic Regression | 0.67 | 0.69 | 0.59 | 0.81 |
| Naïve Bayes | 0.67 | 0.74 | 0.78 | 0.71 |
| kNN | 0.98 | 0.99 | 0.97 | 1.0 |

| Models | Accuracy | F1 | Recall | Precision |
| --- | --- | --- | --- | --- |
| **Random Forest** | **0.72** | **0.76** | **0.73** | **0.79** |
| Decision Tree | 0.93 | 0.94 | 0.89 | 1.0 |
| Multi-Layer Perception | 0.90 | 0.92 | 0.95 | 0.90 |
| C-Support Vector | 0.78 | 0.83 | 0.86 | 0.80 |
| Logistic Regression | 0.68 | 0.73 | 0.70 | 0.76 |
| Naïve Bayes | 0.67 | 0.74 | 0.78 | 0.71 |
| kNN | 0.98 | 0.99 | 0.97 | 1.0 |

**Table S9**: Summary of all the Classification Models When Scoring is Set to F1-score for AMPs targeting *M. tuberculosis* H37Rv and *M. smegmatis* mc2 155 of the “train” set. The Random Forest Classifier, highlighted in bold, is chosen as the best-performing model given its well-balanced performance across different metrics based on its performance on the “test” set (**See Table 6**).

**
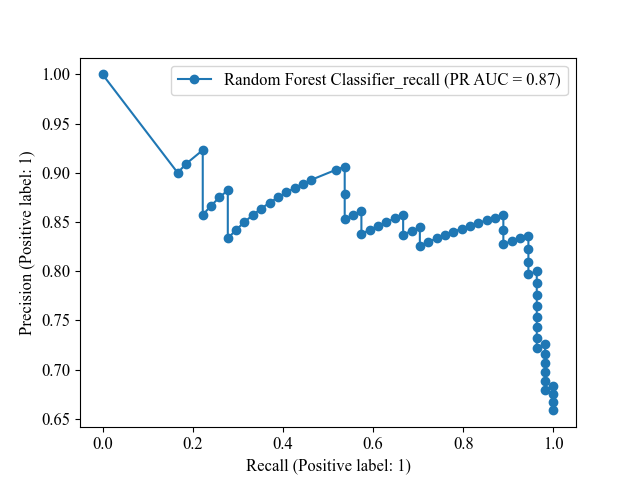
**

**Figure S1:** A precision-recall area under the curve (PR-AUC) of the Random Forest Classifier for the dataset of AMPs targeting [*P. aeruginosa*](https://www.bing.com/ck/a?!&&p=f230aa10195c9d06f193d941708ba1a1dcbe286ac86d39ce7407ca47ecb68d3bJmltdHM9MTc0NTAyMDgwMA&ptn=3&ver=2&hsh=4&fclid=082e62e2-70d8-6464-3b6e-712f71d7658a&psq=PAO1&u=a1aHR0cHM6Ly93d3cubmNiaS5ubG0ubmloLmdvdi9udWNjb3JlL05DXzAwMjUxNg&ntb=1) PAO1, with recall as the optimization metric. The area under the curve (AUC) indicates a PR-AUC value of 0.87, reaffirming the suitability of the Random Forest Classifier for SHAP ananlysis alongside the average precision (AP) value of 0.86 (**See Figure 2**).

**
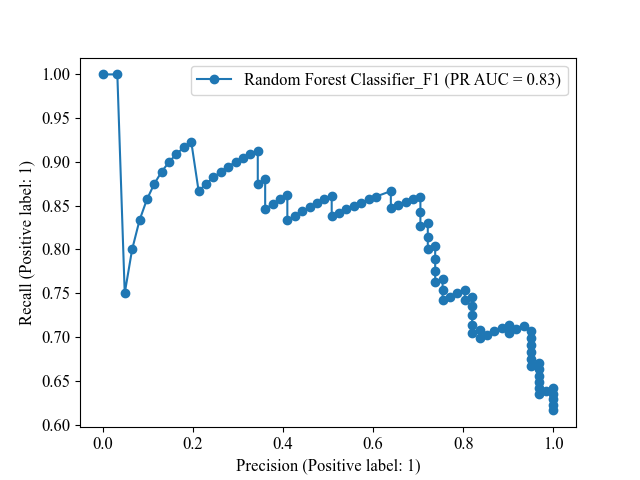
**

**Figure S2:** A precision-recall area under the curve (PR-AUC) of the Random Forest Classifier for the dataset of AMPs targeting [*S. aureus*](https://www.bing.com/ck/a?!&&p=f230aa10195c9d06f193d941708ba1a1dcbe286ac86d39ce7407ca47ecb68d3bJmltdHM9MTc0NTAyMDgwMA&ptn=3&ver=2&hsh=4&fclid=082e62e2-70d8-6464-3b6e-712f71d7658a&psq=PAO1&u=a1aHR0cHM6Ly93d3cubmNiaS5ubG0ubmloLmdvdi9udWNjb3JlL05DXzAwMjUxNg&ntb=1) ATCC 29213, with F1-score as the optimization metric. The area under the curve (AUC) indicates a PR-AUC value of 0.83, confirming the suitability of the Random Forest Classifier for SHAP ananlysis alongside the average precision (AP) value of 0.83 (**See Figure 3**).


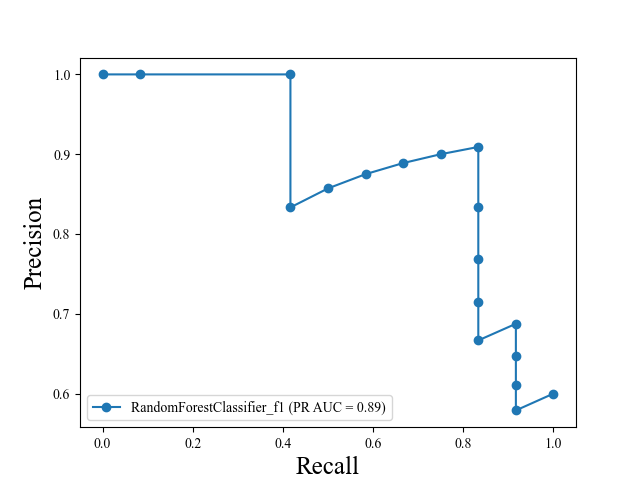


**Figure S3:** A precision-recall area under the curve (PR-AUC) of the Random Forest Classifier for the dataset of AMPs targeting *M. tuberculosis* H37Rv and *M. smegmatis* mc2 155, with F1-score as the optimization metric. The area under the curve (AUC) indicates a PR-AUC value of 0.89, confirming the suitability of the Random Forest Classifier *M. tuberculosis* H37Rv and *M. smegmatis* mc2 155 for SHAP ananlysis alongside the average precision (AP) value of 0.89 (**See Figure 3**).
